# SARS-CoV-2 Persistent Viral Shedding in the Context of Hydroxychloroquine-Azithromycin Treatment

**DOI:** 10.3390/v13050890

**Published:** 2021-05-12

**Authors:** Michel Drancourt, Sébastien Cortaredona, Cléa Melenotte, Sophie Amrane, Carole Eldin, Bernard La Scola, Philippe Parola, Matthieu Million, Jean-Christophe Lagier, Didier Raoult, Philippe Colson

**Affiliations:** 1 Aix Marseille University, IRD, AP-HM, MEPHI, 13005 Marseille, France; clea-leila.melenotte@ap-hm.fr (C.M.); sophie.amrane@ap-hm.fr (S.A.); bernard.la-scola@univ-amu.fr (B.L.S.); matthieu.million@univ-amu.fr (M.M.); jean-christophe.lagier@univ-amu.fr (J.-C.L.); didier.raoult@gmail.com (D.R.); philippe.colson@univ-amu.fr (P.C.); 2 IHU Méditerranée Infection, 13005 Marseille, France; sebastien.cortaredona@univ-amu.fr (S.C.); carole.eldin@ap-hm.fr (C.E.); philippe.parola@univ-amu.fr (P.P.); 3 Aix Marseille University, IRD, SSA, VITROME, 13005 Marseille, France

**Keywords:** SARS-CoV-2, COVID-19, viral persistence, culture, qRT-PCR, hydroxychloroquine, azithromycin

## Abstract

SARS-CoV-2 nasopharyngeal shedding contributes to the spread of the COVID-19 epidemic. Among 3271 COVID-19 patients treated at the Hospital University Institute Méditerranée Infection, Marseille, France from 3 March to 27 April 2020, tested at least twice by qRT-PCR, the median SARS-CoV-2 nasopharyngeal shedding duration was 6 days (range 2–54 days). Compared with short shedders (qRT-PCR positivity < 10 days), 34 (1.04%) persistent shedders (qRT-PCR positivity ≥ 17 days; mean ± SD: 23.3 ± 3.8 days) were significantly older, with associated comorbidities, exhibiting lymphopenia, eosinopenia, increased D-dimer and increased troponin (*p* < 0.05), and were hospitalized in intensive care unit in 17.7% vs. 1.1% of cases (*p* < 0.0001). Viral culture was positive in six persistent shedders after day 10, including in one patient after day 17, and no viral co-pathogen was detected in 33 tested patients. Persistent shedders received azithromycin plus hydroxychloroquine ≥ 3 days in 26/34 (76.5%) patients, a figure significantly lower than in short shedders (86.6%) (*p* = 0.042). Accordingly, mortality was 14.7% vs. 0.5% (*p* < 0.0001). Persistent shedding was significantly associated with persistent dyspnea and anosmia/ageusia (*p* < 0.05). In the context of COVID-19 treatment, including treatment with azithromycin plus hydroxychloroquine, the persistence of SARS-CoV-2 nasopharyngeal shedding was a rare event, most frequently encountered in elderly patients with comorbidities and lacking azithromycin plus hydroxychloroquine treatment.

## 1. Introduction

SARS-CoV-2, responsible for COVID-19, is detected in the nasopharynx, which could constitute a major portal of entry for this emerging pathogen [1]. Indeed, not only is SARS-CoV-2 RNA routinely detected in nasopharyngeal swabs of COVID-19 patients [2], but this clinical material yields living viruses after it is appropriately cultivated in cell culture systems [3,4]. These data suggest that SARS-CoV-2 nasopharyngeal shedding is of interest for the natural history of COVID-19 in patients and in populations, as it may relate to the prognosis of the infection and its contagiousness. Two meta-analyses, including 79 and 28 studies, converged to indicate a viral shedding duration of 17 days (mean) and 18.4 days (median), respectively [5,6].

Monitoring more than 3200 COVID-19 patients firmly documented by real-time RT-PCR (qRT-PCR) at the Hospital University Institute Méditerranée Infection, Marseille, France in March–April 2020 provided the opportunity to clarify the clinical and virological characteristics of patients with persistent nasopharyngeal shedding of SARS-CoV-2 [2].

## 2. Materials and Methods

### 2.1. Patients

This retrospective study aimed to describe the duration of SARS-CoV-2 viral shedding among 3737 qRT-PCR-confirmed COVID-19 patients, followed from 3 March to 27 April 2020 in the Méditerranée Infection Institute, as previously reported [2]. Clinical, radiological and laboratory data were collected, as previously reported [2,7], and the severity of patient illness was evaluated using the National Early Warning Score version 2 (NEWS-2) and the Charlson score [8]. All patients without contraindications were offered oral hydroxychloroquine (HCQ) (200 mg, three times a day for ten days) and azithromycin (AZ) (500 mg at day 1, followed by 250 mg per day for 4 days), and ceftriaxone or ertapenem was added for seven days in case of a NEWS-2 ≥ 5 [9,10]. Initiation of medical care (i.e., medical consultation and initiation of treatment) was defined as day 0, outpatient reevaluation was offered at days 2, 6 and 10 (more if needed), while hospitalized patients were evaluated twice daily. For hospitalized patients, qRT-PCR testing was performed on respiratory samples collected every day after diagnosis. For ambulatory followed-up patients, respiratory samples were collected at days 2, 6, 10 after diagnosis, then every four days when still qRT-PCR-positive at day 10. Biological results used for the analysis were those of tests performed on the day of admission to hospital (day-care hospital or complete hospitalization). The main criteria for discharge from complete hospitalization were the mean (or a significant decrease in flow) of oxygen supplementation

### 2.2. Virology

All patients were SARS-CoV-2-diagnosed based on at least one positive qRT-PCR test performed by nasopharyngeal swab. The time of follow-up was defined relative to the qRT-PCR diagnosis. SARS-CoV-2 genome sequencing was performed directly from nasopharyngeal swab RNA extract using the Illumina MiSeq sequencer and Nextera XT paired-end technology (Illumina, Evry, France) and genome sequences were compared with the reference SARS-CoV-2 isolate Wuhan Hu-1, genome sequence (NC 045512.2) [11]. In case of qRT-PCR cycle threshold (Ct) value >18, partial spike gene PCR amplification (nucleotides 21,296–23,424 in reference to NC 045512.2) was performed, as previously described (DOI: https://doi.org/10.35088/4y1e-ec62, accessed on 11 May 2021). Viral cultures were performed as previously described [3,4]. Briefly, 500 µL of nasopharyngeal swab fluid (Virocult, Elitech, France) or sputum sample were passed through a 0.22-µm pore sized centrifugal filter (Merck millipore, Darmstadt, Germany), then inoculated in 4 wells of 96-well culture microplates containing Vero E6 cells (ATCC CRL-1586) into Minimum Essential Medium culture medium with 4% fetal calf serum and 1% glutamine. All samples were kept for less than 24 h at +4 °C before processing. After centrifugation at 4000× *g*, microplates were incubated at 37 °C. They were observed daily for evidence of cytopathogenic effect. Two subcultures were performed weekly. Presumptive detection of virus in supernatant showing cytopathic effect was done using the SU5000 scanning electron microscope (Hitachi High-Tech Corporation, Tokyo, Japan), before confirmation by a qRT-PCR targeting the SARS-CoV-2 gene E [2]. Culturing was performed systematically for hospitalized patients, and prospectively or retrospectively for non-hospitalized persistent viral shedders. Patients for whom the qRT-PCRs became negative within 10 days were designated as short viral shedders, patients with positive qRT-PCRs between day 10 and 17 as long viral shedders and patients with positive qRT-PCRs after day 17 as persistent viral shedders.

### 2.3. Co-Pathogen Detection

Co-pathogens in the nasopharyngeal swabs were screened by using a multiplex test incorporating 21 targeted pathogens, following the instructions of the supplier (FTD Respiratory pathogens 21; Siemens Fast Track Diagnosis, Luxembourg).

### 2.4. Statistical Analysis

We used the Student *t*-test, Mann–Whitney U test, Chi2 test or Fisher’s exact test to compare differences between short viral shedding and persistent viral shedding groups. To explore risk factors associated with the duration of viral shedding, we performed multivariable analyses using linear regression models. All variables significant at *p* < 0.10 in univariate analyses were introduced in the initial multivariable model. A backward approach was then used to assess the iteration of variables and to control potential confounders (the significance level value for stay was set at 0.05). A primary model was performed over the full sample and a secondary model was performed among patients aged 65 years and older [12]. A secondary analysis using a multivariable logistic regression model was also performed to identify the risk factors associated with prolonged COVID-19 symptoms. The same backward approach was applied to this secondary outcome. A two-sided α of less than 0.05 was considered statistically significant. All analyses were carried out using SAS 9.4 statistical software (SAS Institute, Cary, NC, USA).

### 2.5. Ethics Statement

Data were collected retrospectively from the routine care setting. This non-interventional retrospective study was approved on 13 May 2020 by our institutional review board committee (Méditerranée Infection No.: 2020–021). In compliance with European General Data Protection Regulation No. 2016/679, patients were informed of the potential use of their medical data and that they could refuse the use of their data. The analysis of collected data followed the reference methodology MR-004 registered on No. MR 5010010520 in the AP-HM register, in compliance with European General Data Protection.

## 3. Results

### 3.1. Population Description

Among 3737 COVID-19 patients followed at the Hospital University Institute Méditerranée Infection between March 3 and April 27 [2], 3271 patients had at least two positive SARS-CoV-2 qRT-PCRs performed within less than 10 days. These patients had a median duration of shedding of 6 days (range, 2–54 days): in detail, 2800 (85.6%) patients were short shedders, with a 4.8 ± 3.1 day (mean ± SD) shedding duration; 437 (13.3%) patients were long shedders, with a 15.2 ± 3.8 day shedding duration; and 34 (1.04%) patients were persistent shedders, with a 23.3 ± 3.8 day shedding duration (Table 1). 

Compared to short shedders, persistent shedders were significantly (*p* < 0.01) older (≥65 years old), although the age range was 21–93 years, and 13/34 patients were <45 years old, with age-related comorbidities including chronic heart disease, hypertension, a NEWS-2 ≥5 and a Charlson score ≥5, indicative of an 85% probability of death in 13/34 (38.2%) patients and subsequent hospitalization (Table 1). Furthermore, the initial laboratory check-up of persistent shedders indicated a higher neutrophil/lymphocyte ratio, eosinopenia and higher D-dimer, troponin and C-reactive protein levels compared to short shedders (Table 2).

Multivariable linear regression indicated that the duration of viral shedding was positively associated with NEWS-2, chronic heart disease and eosinopenia <0.04 G/L (Table 3).

### 3.2. Care and Treatment

A higher percentage of persistent shedders was hospitalized in the intensive care unit compared to short shedders, 17.7% vs. 1.1% (*p* < 0.0001). In this cohort, 26/34 (76.5%) persistent shedders had HCQ-AZ therapy ≥ 3 days, versus 2426/2800 (86.6%) short shedders (*p* = 0.042, Mantel–Haenszel Chi-2 test). Indeed, 8/34 persistent shedders had HCQ-AZ therapy < 3 days: more precisely, one patient refused HCQ and AZ treatment, and seven other patients received AZ only because HCQ was contraindicated due to cardiac contraindications (*n* = 6) and drug interaction (*n* = 1) (Table 1). In contrast, 374/2800 short shedders had HCQ-AZ therapy < 3 days (Table 3). Persistent viral shedding was also associated with persistent dyspnea 30 days after the onset of the follow-up. Mortality was 14.7% among persistent shedders vs. 0.5% among short shedders (*p* < 0.0001). At the ~9–10-month follow-up in January 2021, 5/34 persistent shedders had died and seven had been seen in consultation: three patients had persistent post-COVID-19 dyspnea, including one patient who had persistent ageusia. Kinetics analysis showed that eosinophil and lymphocyte counts were significantly lower in patients with persistent viral shedding than in those with short viral shedding, whereas neutrophils were higher in persistent viral shedders. D-dimers and troponin were significantly and persistently higher in persistent viral shedders (Supplementary Table S1).

### 3.3. Viral Genotype Analysis

A total of 21 complete (*n* = 11) or partial (approximately the first half of the spike-encoding gene, *n* = 10) genome sequences were obtained for the 34 persistent shedders, comprising 14 classified in Nextstrain clade 20A, 3 in clade 20B and 4 in clade 20C [13]. This distribution into viral clades did not differ from that observed in non-persistent viral shedders during the same period (310 in clade 20A, 55 in clade 20B and 94 in clade 20C, for a total of 466 genome sequences). All spike sequences harbored the D614G substitution that was present in almost all SARS-CoV-2 genomes in Europe since the epidemic onset. No additional mutations were present within the spike.

### 3.4. Viral Culture

**Figure 1:**
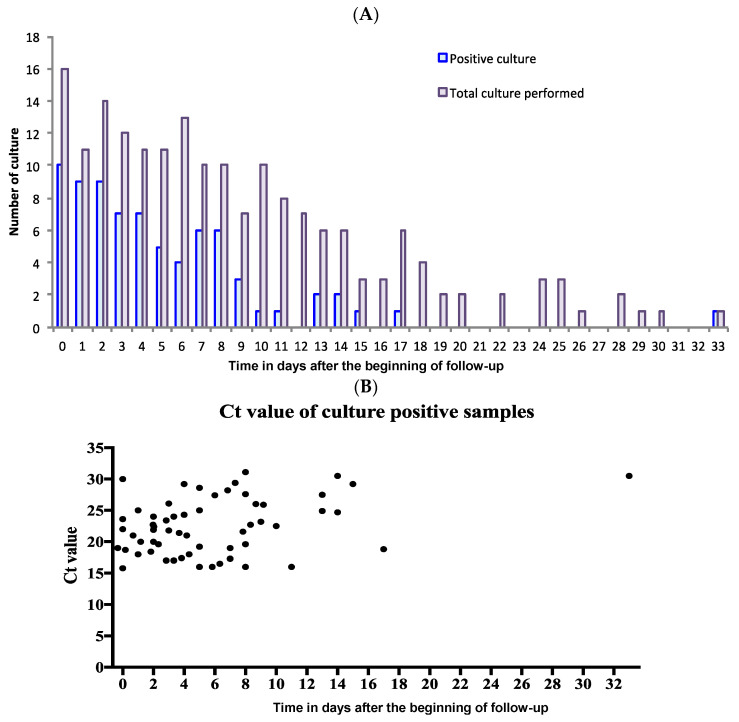
Culture results on 34 patients with persistent viral shedding (>17 days). (**A**) Number of positive cultures for SARS-CoV-2 among the number of cultures performed. (**B**) SARS-CoV-2 cycle threshold values of samples with positive culture. Ct, cycle threshold value; 11 among the 69 samples had positive SARS-CoV-2 culture without Ct value.

A total of 177 nasopharyngeal swabs were cultured in the 34 patients, of which 69 swabs collected in 20 patients were positive in culture. More precisely, eight swabs collected in six patients had a positive culture when collected >10 days after the beginning of follow-up, at days 11, 13, 14, 15, 17 and 33 (Figure 1).

## 4. Discussion

In a large series of qRT-PCR-documented COVID-19 cases followed at the Méditerranée Infection Institute in March and April 2020, the median SARS-CoV-2 nasopharyngeal shedding was six days, shorter than that reported in the literature [14]. Moreover, less than 1% of cases (34/3737) exhibited shedding ≥17 days, and persistent positivity of qRT-PCR in nasopharyngeal swabs correlated with culture positivity. Testing procedures and discharge criteria remained the same during the study period. We observed positive cultures more than 10 days after the onset of the follow-up in eight of 34 patients, a situation rarely reported, as nasopharyngeal shedding is usually reported on the sole basis of qRT-PCR positivity [6]: one case of 4-month viral shedding was reported in the absence of culture and contagiousness [15], whereas anecdotal persistent viral shedding, with positive culture for up to 60 days, has been reported in cancer patients receiving chemotherapy [16].

Previous case reports and a small series of patients experienced prolonged shedding with SARS-CoV-2 (Table 4) [17,18,19,20,21,22,23,24,25,26,27,28,29,30,31,32,33,34,35], mostly involving immunocompromised individuals, including patients diagnosed with hematological malignancies in most cases.

This was not our situation, where only one patient had lymphoma and another had immunosuppression following kidney transplantation. In addition, several cases of viral shedding beyond 90 days occurred in patients who received treatments with convalescent plasma and/or remdesivir [17,18]. These situations were not encountered in this series, as no patient received remdesivir or convalescent plasma and the figures reported here were observed in the context of standardized care, including the prescription of hydroxychloroquine and azithromycin treatment. It is noteworthy that almost one quarter of the 34 persistent shedders reported here did not receive the combination of hydroxychloroquine and azithromycin, a prevalence significantly higher than that in short shedders. This standardized care notably differed in other series reporting a higher proportion of persistent shedders, yet populations may not have been similar, bringing into question the impact of combination of hydroxychloroquine plus azithromycin at the dosage prescribed in reducing the time of SARS-CoV-2 nasopharyngeal shedding.

This reported series mirrors the first COVID-19 epidemic in our region, which was caused by SARS-CoV-2 of clades 20A, 20B and 20C. Accordingly, based on next-generation population sequencing we did not observe any genotype pattern in any of the 21 patients whose viral genome could be explored. Further ultradeep sequencing of paired early and late nasopharyngeal swabs in the 34 patients reported here may nevertheless reveal quasi-species and minority genotypes that may have escaped the standard genotyping methods used in this report.

Whether the data here reported on the genetic context of SARS-CoV-2 circulating in our region one year ago would apply to that of SARS-CoV-2 variants responsible for current epidemics [36] remains to be explored.

## Figures and Tables

**Table 1:** Summary of 34 COVID-19 patients with prolonged SARS-CoV-2 viral shedding ≥ 17 days, Marseille, France, March–April 2020.

Age (years), Gender	Duration of Viral Shedding (days)	Cause of IMMUNODEPRESSION	Serology	NEWS-2 Score	Charlson (Probability of Death in the Following Year)	Treatment	Death (days), COVID-19-Imputable	Viral Genotype
60, M	24	-	+	5	2 (26%)	HCQ-AZ	-	N.a.
41, M	23	-	+	6	2 (26%)	HCQ-AZ	-	20A
21, M	20	-	N.a.	2	0 (12%)	HCQ-AZ	-	20C
41, M	20	-	+	0	1 (26%)	HCQ-AZ	-	20A
30, F	18	-	-	3	0 (12%)	HCQ-AZ	-	N.a.
43, M	19	-	N.a.	4	1 (26%)	HCQ	-	N.a.
37, M	21	-	+	0	0 (12%)	HCQ-AZ	-	20B
37, F	17	-	+	2	0 (12%)	HCQ-AZ	-	20A/25563T/2416T/8371T
74, M	25	-	N.a.	9	8 (85%)	AZ	-	20A
63, M	18	-	-	4	4 (52%)	HCQ-AZ	-	20A
61, F	40	-	+	4	3 (52%)	HCQ-AZ	-	20A
81, M	25	-	-	6	7 (85%)	AZ	-	N.a.
47, F	21	-	+	2	1 (26%)	HCQ-AZ	-	20B
93, F	17	-	N.a.	4	7 (85%)	AZ	-	N.a.
89, F	20	-	+	8	5 (85%)	HCQ-AZ	22, Yes	20A
28, F	26	-	+	2	0 (12%)	HCQ-AZ	-	20A
56, F	54	-	+	2	2 (26%)	HCQ-AZ	-	N.a.
69, M	31	-	N.a.	5	3 (52%)	HCQ-AZ	-	20C
71, M	18	-	+	7	5 (85%)	No HCQ, No AZ	-	N.a.
30, F	21	-	N.a.	5	0 (12%)	HCQ-AZ	-	20A/25563T/2416T/8371T
52, F	20	-	+	4	2 (26%)	HCQ-AZ	-	20A
88, F	17	-	+	7	7 (85%)	AZ	-	20A
70, F	17	-	+	8	6 (85%)	HCQ	289, No	20B
82, M	18	-	N.a.	4	7 (85%)	HCQ-AZ	-	N.a.
43, F	17	-	+	0	1 (26%)	HCQ	-	20C
69, M	19	-	N.a.	6	3 (52%)	HCQ-AZ	-	20A
90, F	25	-	N.a.	6	6 (85%)	HCQ-AZ	51, Yes	20A
76, M	23	-	+	11	5 (85%)	HCQ-AZ	-	N.a.
89, M	21	-	N.a.	12	7 (85%)	HCQ-AZ	-	20C
43, M	19	Lymphoma	+	4	3 (52%)	HCQ-AZ	-	N.a.
73, M	19	-	+	8	7 (85%)	HCQ-AZ	20, Yes	N.a.
64, F	22	-	+	2	3 (52%)	HCQ-AZ	-	N.a.
34, F	23	-	-	2	0 (12%)	HCQ-AZ	-	N.a.
69, F	25	Kidney transplantation	-	3	7 (85%)	HCQ-AZ	-	20A

N.a., not available; HCQ-AZ stands for treatment combining hydroxychloroquine plus azithromycin ≥3 days (refer to text for posology).

**Table 2:** Comparison of biological data for 2800 short shedders (nasopharyngeal SARS-CoV-2 qRT-PCR positivity < 10 days) and 34 persistent shedders (nasopharyngeal SARS-CoV-2 qRT-PCR positivity ≥ 17 days).

	Short Viral Shedders		Persistent Viral Shedders	
	*n* = 2800		*n* = 34	
Biological Data	N	Mean (std)	N	Mean (std)
Age (years)	2800	44.2 (15.9)	34	58.4 (20.9)
Lymphocytes (G/L)	2358	1.44 (0.64)	31	1.05 (0.47) *
Neutrophils (G/L)	2285	3.37 (1.8)	27	4.42 (2.6)
Neutrophils/lymphocytes	2285	2.84 (3.19)	27	4.71 (3.44) *
Eosinophils (G/L)	2347	0.08 (0.09)	31	0.04 (0.06) *
D dimers (µg/mL)	457	0.99 (2.19)	19	1.25 (1.01) *
Troponin (ng/L)	246	9.99 (12.18)	16	18.71 (18.79) *
CRP (mg/L)	2130	16.31 (34.6)	31	31 (48.09) *

CRP, C-reactive protein; * denotes statistical significance using the Chi-square, Fisher’s exact or Wilcoxon–Mann–Whitney test where appropriate and a 0.05 *p* value. See Supplementary Table S1 for the complete list of data.

**Table 3:** Risk factors associated with duration of viral shedding as assessed by multivariable linear regression.

All (*n* = 2378 *)	Beta 95% Confidence Interval	*p*-Value
Time between symptom onset and treatment onset (days)	−0.16 (−0.20; −0.13)	<0.0001
NEWS-2 ≥ 5	0.78 (0.16;1.41)	0.0139
Symptoms of COVID-19 at day 0 (reference = none)	1.37 (0.83;1.91)	<0.001
Chronic heart disease (reference = none)	1.01 (0.28;1.74)	0.0065
Eosinophils < 0.04 G/L (reference >0.04 G/L)	0.83 (0.51;1.16)	<0.001
**Patients aged 65 and older (*n* = 294)**	**Beta 95% confidence interval**	***p*-value**
Time between symptom onset and treatment onset (days)	−0.29 (−0.45; −0.14)	0.0002
Other treatment (reference= HCQ + AZ ≥ 3 days)	2.39 (0.90; 3.87)	0.0016

* 456 patients with missing eosinophils data were excluded from the model. Risk factors were selected using backward selection with alpha = 0.05.

**Table 4:** Epidemiological, virological and clinical features of cases of prolonged SARS-CoV-2 infections in immunocompromised patients.

Reference	Gender, Age (Years)	Immunodepression Cause	Duration of Viral Shedding ^a^	Remdesivir	Convalescent Plasma or anti-Spike Antibodies	Other Therapies(s)	Number of Amino Acid Substitutions/Deletions in the Genome and/or in the Spike Protein	Outcome
[17]	Male, 45 y.-o.	Severe antiphospholipid syndrome	151 days	Days 0–4, 72–81, 105–109, 151–155	ASA: day 143	Glucocorticoids, cyclophosphamide, intermittent eculizumab and rituximab, ruxolitinib	24 substitutions, 3 deletions (spike: 12 substitutions, 1 deletion) among which substitution N501Y present in variants 20I/501Y.V1, 20H/501Y.V2 and 20J/501Y.V3 ^b^, and E484K present in variants 20H/501Y.V2 and 20J/501Y.V3 ^b^	Death on day 154
[18]	Not reported	Marginal B cell lymphoma (received B cell depletion therapy; hypogamma-globulinemia)	101 days	Days 41-, 54-, 93-	CP: days 63, 65, 95	-	Spike: 5 substitutions among which N501Y and deletion H69/V70 both present in 20I/501Y.V1 ^b^	Not reported
[19]	Male, 60 y.-o.	Mantle cell lymphoma	156 days	Days 30-, 122-	CP: days 33, 122	CD20 bispecific antibody, second B-cell directed antibody, cyclophosphamide, doxorubicine, prednisone	6 substitutions	Pursued home hospice care
[20]	Male, 75 y.-o.	Multiple myeloma	71 days	Days 5-9	CP: days 2, 58	Dexamathasone (days 63–74)	Spike: 9 substitutions between days 4 and 67, including D215G present in 20H/501Y.V2 ^b,^ Y144 deletion present in 20I/501Y.V1 ^c^, and N501T at a position mutated in variants 20I/501Y.V1, 20H/501Y.V2 and 20J/501Y.V3 ^b^	Death on day 74
[21]	Male, 60-70 y.-o.	Non-Hodgkin lymphoma	268 days	Days 47-51, 77–86, 178–182, 205–209	CP: day 88	Darunavir/ritonavir, hydroxychlorquine, IV methylprednisolone, tocilizumbab, ceftaroline	26 substitutions; spike: 7 including H69Y/P and V70G at positions mutated in variant 20I/501Y.V1 ^b^	Death on day 271
[22]	Female, 53 y.-o.	Follicular lymphoma	85 days	Days 63–72, 80–84	CP: day 85 ^c^	-	No genome sequencing reported	
[23]	Female, 17 y.-o.	Pre-B-cell acute lymphoblastic leukemia	100 days	Days 13–22, days 60–69	CP: day 61	Hydroxychloroquine for two days; methylprednisolone	No genome sequencing reported	qRT-PCR-positive on day 100; no supplemental oxygen
[24]	Male, 50-60 y.-o.	Chronic lymphocytic leukemia	63 days	Days 23–33, days 45–55	CP: day 58	-	No genome sequencing reported	
[25]	Female, 41 y.-o.	Severe hypogammaglobulinemia	75 days	No	CP: days 71, 72	Prednisone	No genome sequencing reported	Discharge
[25]	Male, 65 y.-o.	Common variable immunodeficiency	40 days	No	No	Lopinavir/ritonavir, broad-spectrum antibiotics	No genome sequencing reported	Death on day 40
[26]	Female, 70-79 y.-o.	Follicular lymphoma	>134 days	No	CP: ≈days 45, 65, 95, 110, and 115	Steroids	24 substitutions, 2 deletions; spike: 3 substitutions including E484K present in variants 20H/501Y.V2 and 20J/501Y.V3 ^b^; one deletion Y144 present in variant 20I/501Y.V1 ^c^	Death on day 156
[26]	Not reported	B-cell depleted lymphoma	91 days	No	No	N.a.	At day 19 post-diagnosis: 2 substitutions	Recovery
[27]	Female, 71 y.-o.	Chronic lymphocytic leukemia, hypogammaglobulinemia	105 days	No	CP: days 70, 80	-	6 substitutions and 1 deletion on day 49; spike: 2 substitutions; 3 additional substitutions (2 at day 70, 1 at day 85) and one additional deletion on day 70 in the spike	N.a.
[28]	Female, 47 y.-o.	Follicular lymphoma	59 days	No	No	Obinutuzumab bimonthly, acyclovir, atovaquone, favipiravir, ciclesonide, lopinavir/ritonavir	No genome sequencing reported	Discharge on day 69
[29]	Not reported, median (range), 58 y.-o. (35–77)	Hematological malignancies (*n* = 15); multiple sclerosis (1); common variable immune deficiency (1)	17 patients (median duration = 56 days; max.= 83 days)	N = 3	No	Anti-CD20 monoclonal antibodies (*n* = 15); steroids (8); hydroxychloroquine (*n* = 5); tocilizumab (*n* = 4); lopinavir/ritonavir (*n* = 2)	No genome sequencing reported	One death
[30]	Male, 66 y.-o.	HIV infection (CD4 cell count= 0/mm^3^)	123 days	No	No	Multi-antiretroviral therapy	1 substitution, in the spike	Neurological degradation
[30]	Male, 71 y.-o.	Heart transplantation, diabetes mellitus	121 days	No	No	Prednisone, mycophenolic acid, belatacept	No occurrence of substitutions	N.a.
[30]	Male, 35 y.-o.	Rheumatoid arthritis	84 days	No	No	Rituximab	Occurrence of 6 substitutions, 1 the spike	Improvement
[31]	Female, 5 y.-o.	Dermatomyositis	35 days	No	No	Prednisolone	No genome sequencing reported	Resolution
[32]	Female, 60 y.-o.	Rheumatoid arthritis	>35 days	Day ≈30	CP: Week 5	Rituximab	No genome sequencing reported	Discharge
[33]	Female, 17 y.-o.	Previously healthy	97 days	No	No	Hydroxychloroquine for 5 days	Coinfection with two SARS-CoV-2 lineages (20A, 20B)	N.a.
[34]	Male, 61 y.-o.	Liver transplant	Negative on days 35 and 39, then positive again on days 41 and 48	No	No	Tacrolimus, lopinavir/ritonavir, amoxicillin, piperacillin sulbactam, Lianhua Qingwen	No genome sequencing reported	Discharge on day 55
[35]	Male, 31 y.-o.	X-linked agamma- globulinaemia	62 days (in sputum; 36 days in nasopharyngeal samples)	Days 34–43, 61–70	CP: days 69, 70	Hydroxychloroquine/ azithromycine, meropenem, ceftriaxone, clarithromycin	5 substitutions; spike: 1 substitution	Discharge on day 73

^a^ As assessed by qRT-PCR; ^b^ 20I/501Y.V1 = “UK” variant, 20H/501Y.V2 = “South African” variant and 20J/501Y.V3 = “Brazilian” variant; ^c^ at the end of second cure of remdesivir. ASA: anti-spike antibodies; CP: convalescent plasma; PML: progressive multifocal leukoencephalopathy.

## Data Availability

Data are available from the corresponding author upon reasonable request.

## Supplementary Materials

The following are available online at https://www.mdpi.com/1999-4915/13/5/890/s1, Table S1: Comparison of epidemiological, clinical and biological data for 2800 short shedders (nasopharyngeal SARS-CoV-2 qRT-PCR positivity < 10 days) and 34 persistent shedders (nasopharyngeal SARS-CoV-2 qRT-PCR positivity ≥ 17 days).
